# Assessing muscle energy technique and foam roller self-myofascial release for low back pain management in two-wheeler riders

**DOI:** 10.1038/s41598-024-62881-8

**Published:** 2024-05-27

**Authors:** Tabassum Khan, Moattar Raza Rizvi, Ankita Sharma, Fuzail Ahmad, Shahnaz Hasan, Shadab Uddin, Mohammad Sidiq, Areej Ammari, Amir Iqbal, Ahmad H. Alghadir

**Affiliations:** 1 Department of Physiotherapy, Faculty of Allied Health Sciences, Manav Rachna International Institute of Research and Studies (MRIIRS), Faridabad, 121001 India; 2 School of Allied Health Sciences, Manav Rachna International Institute of Research and Studies (MRIIRS), Faridabad, 121001 India; 3 Respiratory Care Department, College of Applied Sciences, AlMaarefa University, 13713 Riyadh, Saudi Arabia; 4 Department of Physical Therapy and Health Rehabilitation, College of Applied Medical Sciences, Majmaah University, 15431 Al-Majmaah, Saudi Arabia; 5 Department of Physical Therapy, Faculty of Applied Medical Sciences, Jazan University, Jazan, Kingdom of Saudi Arabia; 6 Department of Physiotherapy, School of Medical and Allied Health Sciences, Galgotias University, Greater Noida, Uttar Pradesh India; 7 Department of Medical Rehabilitation, Abu-Arish General Hospital, Jazan, Saudi Arabia; 8 Rehabilitation Research Chair, Department of Rehabilitation Sciences, College of Applied Medical Sciences, King Saud University, 11433 Riyadh, Saudi Arabia

**Keywords:** Muscle energy technique, Self-myofascial release, Foam roller, Hamstring flexibility, Dynamic balance, Physical disability, Sit and reach test, Active knee extension, Rehabilitation, Musculoskeletal system, Occupational health

## Abstract

Pain in the lower back is a major concern in today’s era due to prolonged sitting in two-wheeler riders, mainly due to hamstring tightness. It also creates physical disability and impairment in activities of daily living. The study aimed to compare the efficacy of muscle energy technique (MET) and self-myofascial release (SMFR) using the foam roller on hamstring flexibility, dynamic balance, and physical disability amongst two-wheeler riders with chronic low back pain (LBP). Participants were randomized into two intervention groups, MET and SMFR using the envelope method, with each group having 20 participants. Hamstring flexibility and range of motion for knee extension and the lower back were assessed using the active knee extension test (AKE-L and AKE-R) and sit and reach test (SRT), while the dynamic balance was assessed by the star excursion balance test (SEBT) and physical disability by Roland‐Morris Disability Questionnaire, (RMDQ). Measurements were taken at baseline and after 4 weeks of intervention. This study demonstrated that both SMFR using a foam roller and MET are effective in enhancing hamstring muscle flexibility, (SRT—F(1, 38) = 299.5, p < 0.001; AKE-R—F(1, 38) = 99.53, p < 0.001; AKE-L—F(1, 38) = 89.67, p < 0.001). Additionally, these techniques significantly improved dynamic balance in various directions, including anterior (ANT), anteromedial (AMED), medial (MED), posteromedial (PMED), posterior (POST), posterolateral (PLAT), lateral (LAT), and anterolateral (ALAT) directions (p < 0.01). Furthermore, there was a significant reduction in physical disability (RMDQ—F(1, 38) = 1307, p < 0.001), among two-wheeler riders suffering from chronic LBP. Compared to MET, SMFR using foam rollers was found to be more effective in enhancing hamstring flexibility, improving balance, and decreasing disability level on the RMDQ after 4 weeks.

## Introduction

Most people have back problems at some point in their lives^1^. Low back pain (LBP) is a well-documented, widespread health issue, yet its burden is frequently deemed negligible^2^. The most prevalent type of back pain is mechanical. Mechanical LBP is either specific or non-specific. LBP can be acute (sudden onset less than 6 weeks), subacute (6–12 weeks), chronic (lasting 12 weeks or more)^3^ or relapsing (relapsing after a rest period), depending on the duration. Mechanical or non-specific LBPs are most commonly reported among the motorcycle population^4^. The middle class developing countries considers motorcycles their primary mode of mobility due to their cheap fuel consumption, low maintenance, and ease of commuting in congested locations^5^. Despite these benefits, bicycle riding is a complicated and somewhat hazardous activity^6^. Humans generate a vast array of postural responses in response to balance disruptions while driving a two-wheeler. Being a postural muscle that maintains balance, the hamstring is one of the most important muscles in the hip strategy^7^. Thus, any malfunction of this muscle can influence balance while using a bike. It has also been found that hamstring muscle strain is one of the risk factors leading to low back discomfort in patients, particularly those who drive constantly for hours^8^. The flexibility of the hamstring when bending forward affects the angle and range of motion (ROM) of the pelvis and thoracic region^9^. Higher levels of disability and physical function impairment, sick leave and work loss, higher healthcare consumption and treatment expenses, and other concurrent conditions have been widely reported in association with chronic LBP (CLBP)^10^. Several studies highlighted the prevalence of low back pain (LBP) in motorcyclists. A study in Pakistan reported that 52.5% of bike riders suffered from LBP^4^. In Ilesa, Nigeria, 41% of commercial motorcyclists experienced LBP^11^, while a rural hospital study in Nigeria found a 46% prevalence of LBP among staff, with drivers exhibiting a 20% rate^12^. Additionally, research in India focusing on non-occupational motorcyclists revealed a 65% incidence of discomfort in the lower back region^13^. These findings suggest a notable prevalence of back pain among two-wheeler riders, though specific data on hamstring pain remains limited.

The pressure on the discs in the lower back could be more pronounced during periods of sitting compared to standing, and since motorcyclists spend most of their time seated while riding^14^, extended sitting could potentially lead to hamstring strain^15^. Consequently, individuals may have an increased likelihood of experiencing lower back pain (LBP). However, it is also essential to acknowledge that not all two-wheeler riders will experience low back pain solely due to prolonged sitting. Individual variations, physical fitness, age and time for which they drive the vehicle and other factors play a role in determining who is more susceptible to this issue. The objectives of physiotherapy for individuals with LBP include pain alleviation, recovery of lost range of motion, restoration of function, and enhancement of quality of life. Several workout regimens, electrotherapy, and relaxation protocols are utilized to accomplish these objectives^16^.

Muscle energy technique (MET) is one of the most frequently used treatment strategies to improve the suppleness of contractile and non-contractile tissues. MET is an osteopathic tissue manipulation technique involving precision-directed and controlled active isometric and isotonic contractions to enhance musculoskeletal function and reduce pain. MET can be used to lengthen and strengthen muscles, improve fluid dynamics, minimize surrounding edema, and mobilize a restricted joint^8^. In addition, the self-assisted myofascial release technique (SMFR) is another treatment choice used by most physiotherapists. SMFR is a part of manual therapy, which is based on the outcomes of applying mechanical pressure to soft body tissue. The mechanism of SMFR is the same as that might be induced during self-massage, and so both words are commonly used interchangeably. This refers to SMFR types that use rollers (foam rolling). The rollers are utilized to extend and provide pressure to the fascia. This encourages histological tissue changes in the treated area, which has suffered pathological changes because of strain, traumatic motions, metabolic imbalance, or even psychological factors. Self-remedy is meant to result in removing signs and symptoms called fascial regulations and adhesions, including pain and reduced ROM^17^.

Individuals who frequently use two-wheelers (motorcycles or bicycles) for more than 2–4 h/day tend to spend extended periods seated on their bikes^4^. This prolonged sitting position can lead to a tightening of the hamstring muscles, which may result in discomfort or pain in the lower back area. Prolonged periods of sitting can contribute to hamstring strain primarily through a mechanism known as adaptive shortening. When seated, the knees are bent, and the hamstrings are shortened. Over time, this sustained position can lead to the muscles adapting to this length, becoming tighter and losing flexibility. This state of chronic shortening and reduced flexibility increases the risk of strain as the muscle becomes less capable of tolerating stretching and sudden movements. Furthermore, sitting for extended periods can also impair blood flow to the hamstring muscles, leading to stiffness and increasing the risk of injury. Thus, even though the hamstrings are not actively engaged while sitting, the combination of adaptive shortening, reduced flexibility, and decreased circulation sets the stage for potential strain when these muscles are eventually called into action during physical activities. The existing literature extensively explores various physiotherapy techniques for managing CLBP, particularly in general populations. However, there is a notable gap in research specifically targeting individuals who frequently use two-wheelers, such as motorcycles or bicycles. While previous research has separately highlighted the benefits of MET and SMFR, there is a lack of comprehensive comparative studies focusing on these methods' impact on hamstring flexibility, dynamic balance, and physical disability.

This study aimed to evaluate and compare the effectiveness of MET and SMFR using foam rollers in two-wheelers with CLBP.

## Materials and methods

### Study design

This study was a two-arm, parallel-group, randomized trial in which all participants diagnosed with chronic low back pain (CLBP) were included. We adhered to the CONSORT (Consolidated Standards of Reporting Trials) reporting guidelines to ensure transparent and comprehensive reporting of the trial design, participant enrollment, interventions, and outcomes.

### Ethical consideration

All procedures involving human subjects in this study were approved by the ethical committee at the Faculty of Allied Health Sciences (Approval No: MRIIRS/FAHS/PT/2022-23/M-010; dated: 04/02/2022) and registered on ClinicalTrial.gov PRS under identifier number: NCT06017804; dated: 30/08/2023. The study was performed in accordance with the Declaration of Helsinki (1975). Before any data was collected, participants were given a thorough description of the study's protocols and sought their written consent.

### Sample size

An a priori power analysis was used to determine the sample size for this study. To determine the appropriate sample size, the G*Power software (ver. 3.1.9.2, Heinrich Heine-University, Düsseldorf, Germany) was used. A repeated measure test was used to compare the between within interaction (MANOVA) of two groups with a power level of 80%, a significance level of 5%, and an effect size of 0.40 from one of the previous study^18^. Based on the above assumptions, the sample size required for this study was 52.

### Eligibility criteria

#### Inclusion criteria

The study targeted two-wheeler riders, both male and female, aged 20–40 years, who rode at least two to four hours daily for the past two years. This group was chosen for their increased risk of lower back issues due to prolonged riding. Participants having CLBP of at least 3–5 on the Visual Analogue Scale radiating to the legs for at least three months, and a specific physical limitation of 15–20-degree loss in knee extension were included. Additionally, they should not have been receiving any ongoing lower back pain treatment.

#### Exclusion criteria

Pregnant women, individuals with recent fractures or surgeries in the spine or lower limbs, and those with previous hamstring injuries were excluded to avoid confounding factors. Participants with lumbar or lower limb neurological deficits, limb length disparity, or long-term medications like corticosteroids and painkillers affecting musculoskeletal symptoms were also excluded. Additionally, those with severe cardiovascular conditions, mental health disorders, ongoing physical therapy, known allergies to intervention materials, and cognitive impairments that could impede informed consent were not considered.

### Participants

**Figure 1:**
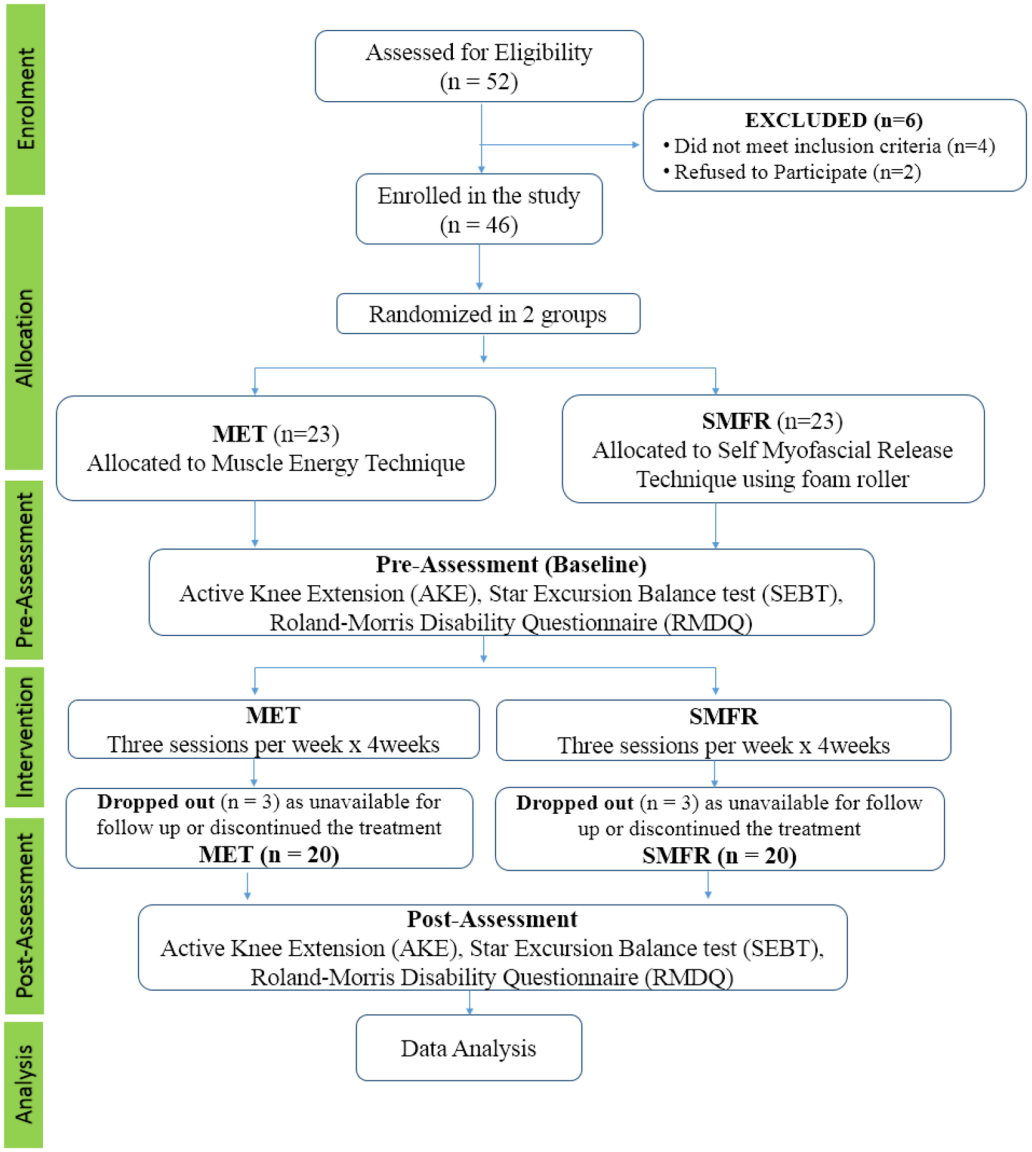
Flow chart of the study design.

The recruitment of participants and pre-and-post-interventional data collection was completed within 8.5 months (from 14/03/2022 to 29/11/2022). Figure 1 depicts the study’s procedures, including the participants’ enrollment, group allocation, pre-assessment, intervention, post-assessment, and data analysis. Fifty-two motorcyclists were screened for CLBP in the National Capital Region (India). Six motorcyclists were excluded during the initial evaluation after failing to meet the eligibility criteria. The remaining 46 motorcyclists were randomly allocated into two intervention groups, Muscle Energy Techniques (MET) and (self-myofascial release) SMFR technique.

### Procedure

Randomization was done using a 1:1 allocation ratio and a concealed envelope. To maintain the integrity and minimize potential bias in this study, a single-blinding procedure was employed. The participants and the two assistant physiotherapists responsible for conducting measurements at baseline and after four weeks of intervention were kept unaware of the participants' group assignments. This single-blinding approach enhanced the objectivity of data collection and reduced the risk of any conscious or subconscious influence on the study outcomes. Single blinding is considered an effective method to minimize bias in randomized trials where the nature of the intervention prevents blinding of the therapists.

Written informed consent was obtained from all the participants. Physical assessment, hamstring flexibility (active knee extension, AKE test)^18^, range of motion for the back and thigh (Sit and Reach Test, SRT)^19^, dynamic balance (star excursion balance test, SEBT)^20^ and physical disability (Roland-Morris Disability Questionnaire, RMDQ)^21^. Two assistant physiotherapists took the measurements at baseline and after four weeks of intervention, and the participants were kept blind to the participant’s group allocation. Further, six participants (three from each group) dropped out of the study at different time points during the follow-up of 4 weeks. Therefore, the post-measurement of outcome variables was done on 20 participants in each group.

Participants were provided with a comprehensive explanation of the potential risks associated with enrolling in the study and undergoing the specific intervention. They were encouraged to promptly report any complaints or discomfort they experienced during the research to ensure their safety and well-being throughout the study.

### Outcome measures

#### Hamstring flexibility assessment: active knee extension (AKE) test

The Active Knee Extension (AKE) test^18^ was employed to quantify the extensibility of the hamstring muscle group. Participants were positioned in a supine orientation on a treatment table, with the hip joint positioned in 90 degrees of flexion to isolate the biarticular hamstring musculature spanning the hip and knee joints. The contralateral lower extremity remained in an extended position on the treatment table. Participants were instructed to actively extend the knee joint until they reported the onset of a stretching sensation in the posterior thigh region, without eliciting discomfort or pain. A digital goniometer was carefully aligned with the anatomical landmarks of the lateral femoral condyle and lateral malleolus to accurately measure the knee extension angle, providing a precise assessment of the length of the hamstring muscle group.

#### Range of motion assessment: sit and reach test (SRT)

The Sit and Reach Test (SRT)^19^ was administered to evaluate the flexibility of the posterior kinetic chain musculature, encompassing the hamstring and lumbar erector spinae muscle groups. Participants were instructed to assume a seated position on the floor with their backs against a wall and lower extremities extended, with the soles of their feet positioned flat against a standardized sit-and-reach box. Participants were then instructed to reach forward with both upper extremities in a controlled manner, without flexing the knees, and to hold the maximum reach position for 2 s. The distance between the tip of the extended fingers and the edge of the box was recorded using a calibrated measuring scale affixed to the box, offering a direct quantification of the flexibility of the posterior kinetic chain, including the hamstring and lumbar spine musculature.

#### Dynamic balance assessment: star excursion balance test (SEBT)

Dynamic balance and proprioceptive control were comprehensively assessed through the Star Excursion Balance Test (SEBT)^20^. Participants maintained a unilateral stance while standing in the center of a grid comprised of eight directional vectors (anterior, anterolateral, lateral, posterolateral, posterior, posteromedial, medial, and anteromedial) marked on the floor. Participants were instructed to reach as far as possible with the contralateral lower extremity along each directional vector while maintaining their balance on the stance limb. The maximal reach distance achieved in each direction was quantified using a measuring tape affixed to the floor, and was normalized to the participant's leg length to account for anthropometric differences. This test challenged the intricate interplay between dynamic stability, proprioceptive acuity, neuromuscular control, and lower extremity function. The normalized reach distances in each direction were utilized to evaluate the participant's dynamic postural control and proprioceptive capabilities.

#### Physical disability measurement: Roland–Morris disability QUESTIONNAIRE (RMDQ)

The Roland-Morris Disability Questionnaire (RMDQ)^21^ was employed to measure the degree of physical disability resulting from low back pain. This self-administered, validated questionnaire comprised 24 items evaluating the functional impact of low back pain on various activities of daily living, such as dressing, lifting, walking, and sleeping. Participants were instructed to respond to each item by indicating whether the statement accurately described their current condition or not. Responses were quantified using a dichotomous scoring system (0 or 1), generating a cumulative score ranging from 0 (no disability) to 24 (maximum disability), with higher scores reflecting greater severity of disability and functional impairment associated with low back pain.

### Experimental intervention

#### Muscle energy technique (MET)

Participants were required to adopt a supine position. MET's post-isometric relaxation method was chosen. The therapist gently flexed the patient's hip until the limitation barrier was detected. At this stage, isometric contractions against resistance were applied. After that, the patients were told to resist the movement based on their own perception and comfort level. Before the leg was released, the contraction was maintained for 7–10 s. On exhale, the knee joint was straightened (extended) towards its new barrier, and a stretch was applied and maintained through that barrier for 30 s. This procedure was carried out six times. The intervention lasted four weeks and consisted of three weekly training sessions, each session lasting for about 30 minutes^22^.

#### Self-myofascial release (SMFR) using foam roller

A foam roller was rolled from the ischial tuberosity to the back of the knee on the side being tested while the participant remained seated in a prolonged sitting position. Individuals were instructed to keep all of their body weight on the leg being evaluated. They spent 30–40 s rolling one hamstring at a time in each set (10 times back and forth). The intervention period was performed on both legs and consisted of three training sessions per week for four weeks, each session of about 20 min. A hard type 66 × 14 cm foam roller, made of Ethylene Vinyl Acetate Copolymer, was used as the SMFR tool^17^.

### Statistical analysis

Statistical analysis was done using the statistical package for social science (SPSS) version 23 (IBM Corp., Armonk, NY, USA). In this study, normality and variance homogeneity were determined using the Shapiro–Wilk and Levene tests and the results of both tests were greater than 0.5. Data are presented as means ± standard deviation (SD). The differences within groups were compared using paired t-tests. Differences between groups were evaluated using unpaired (independent) t-tests. A 95% confidence interval was computed for all correlation coefficients. The two-way repeated measure ANOVA test was used to compare the effects of the interventions over time and between groups. Cohen's d was calculated to estimate the effect size, with values interpreted as follows: 0.2 to 0.49 indicates a small effect, 0.5 to 0.79 indicates a medium effect, and 0.8 and above indicates a large effect. An alpha level of p ≤ 0.05 was used to determine statistical significance in this study.

## Results

Initially, 52 individuals were assessed for eligibility. Out of these, 6 were excluded; 4 did not meet the inclusion criteria, and 2 declined to participate. Consequently, 46 participants were enrolled and randomized into two groups: 23 to the Muscle Energy Technique (MET) group and 23 to the Self-Myofascial Release Technique using a foam roller (SMFR) group. Over the course of the study, 3 participants from each intervention group were unable to complete the study, amounting to a total of 6 dropouts. This resulted in a dropout rate of approximately 13% per group, calculated from the initial number of participants who started the intervention phase. Reasons for dropout included unavailability for follow-up and personal decisions to discontinue the treatment. With these considerations, the final number for analysis was 20 participants in each group.

There was no significant difference in age, height, body weight, or body mass index (BMI) between the groups receiving Muscle Energy Techniques (MET) and those receiving Self-Myofascial Release (SMFR) (Table 1).

**Table 1:** Independent sample t-test to compare the baseline measurement for hamstring flexibility and physical disability in two groups.

Outcome variables	Time	SMFR Mean ± SD	MET Mean ± SD	MD	t value	p/χ^2^ value	d	95% CI	
								Lower	Upper
Gender (M/F)	–	19/4	20/3	–	0.67	0.70	–	–	–
Age (years)		35.61 ± 2.56	36.21 ± 1.87	0.60	0.91	0.37	0.14	− 0.73	1.93
BMI (kg/m^2^)	–	21.84 ± 0.42	22.05 ± 0.35	0.21	1.842	0.07	0.15	− 0.02	0.44
History of bike riding (hours/week)	–	7.69 ± 0.81	8.07 ± 0.64	0.38	1.77	0.08	0.36	− 0.05	0.81
SRT (cm)	Pre (n = 23)	7.29 ± 2.61	5.78 ± 2.65	1.52	1.82	0.08	0.58	− 0.17	3.20
	Post (n = 20)	9.32 ± 2.58	7.84 ± 2.43	1.48	1.86	0.07	0.59	− 0.13	3.08
AKE-R (°)	Pre (n = 23)	43.5 ± 9.61	44.40 ± 7.71	− 0.90	− 0.34	0.74	− 0.11	− 6.33	4.53
	Post (n = 20)	53.45 ± 8.98	52.20 ± 5.91	1.25	0.52	0.61	0.16	− 3.61	6.11
AKE-L (°)	Pre (n = 23)	43.05 ± 8.13	46.85 ± 8.37	− 3.80	− 1.46	0.15	− 0.46	− 9.08	1.48
	Post (n = 20)	54.05 ± 8.29	55.65 ± 6.82	− 1.60	− 0.67	0.51	− 0.21	− 6.46	3.26
RMDQ	Pre (n = 23)	12.65 ± 1.63	12.15 ± 1.46	0.50	1.02	0.31	0.32	− 0.49	1.49
	Post (n = 20)	3.55 ± 1.05	3.85 ± 1.27	− 0.30	− 0.82	0.42	− 0.26	− 1.05	0.45

*SRT* sit and reach test, *AKE-R* active knee extension of right leg, *AKE-R* active knee extension of left leg, *RMDQ* Roland–Morris disability questionnaire, *M* male, *F* female, *MD* mean difference, *d* effect size (Cohens).

An Independent sample t-test was performed to compare the effect of MET and SMFR on hamstring flexibility and physical disability using the SRT (sit and reach test), the AKE-L and AKE-R (active knee extension left and right) test, and the RMDQ (Roland-Morris disability questionnaire). There was no statistically significant difference in SRT, AKE-L, AKE-R, and RMDQ at pre- and post-assessment (Table 1).

To compare the effects of MET and SMFR on dynamic balance using the Star excursion balance test (SEBT), an independent t-test was used. At pre-assessment, no significant difference was observed in the different directions of the dynamic balance of the right leg except on the posterior side. Post-measurement of the dynamic balance of the right leg in all directions was found to be non-significant (Table 2).

**Table 2:** Independent sample t-test for dynamic balance.

SEBT	Pre	SMFR (n = 23) Mean ± SD	MET (n = 23) Mean ± SD	p-value	t-value	d
Right pre (cm)	ANT	77.18 ± 10.69	83.54 ± 13.30	0.14	2.34	− 0.52
	AMED	78.69 ± 12.04	86.14 ± 13.56	0.40	0.73	− 0.58
	MED	77.46 ± 10.86	84.60 ± 13.52	0.15	2.12	− 0.58
	PMED	76.19 ± 15.08	83.75 ± 16.95	0.53	0.41	− 0.47
	POST	73.93 ± 16.3	82.24 ± 16.41	0.83	**0.05**	− 0.5
	PLAT	71.30 ± 16.81	77.11 ± 19.287	0.47	0.54	− 0.32
	LAT	66.46 ± 13.16	68.91 ± 17.3	0.21	1.65	− 0.15
	ALAT	73.11 ± 14.63	78.78 ± 17.06	0.41	0.70	− 0.35
Left pre (cm)	ANT	79.09 ± 11.00	85.69 ± 12.25	0.57	0.33	− 0.56
	AMED	78.80 ± 12.43	86.32 ± 14.12	0.46	0.57	− 0.56
	MED	77.19 ± 13.82	85.16 ± 12.55	0.59	0.30	− 0.6
	PMED	78.44 ± 14.2	82.72 ± 14.85	0.63	0.23	− 0.29
	POST	75.43 ± 17.21	80.69 ± 14.92	0.38	0.80	− 0.32
	PLAT	74.18 ± 16.53	79.30 ± 17.7	0.66	0.20	− 0.29
	LAT	66.09 ± 12.23	71.07 ± 13.8	0.20	1.73	− 0.38
	ALAT	77.55 ± 14.18	82.78 ± 15.46	0.39	0.76	− 0.35

SEBT	Post	SMFR (n = 20) Mean ± SD	MET (n = 20) Mean ± SD	p-value	t-value	d
Right post (cm)	ANT	87.78 ± 10.80	86.14 ± 12.83	0.19	1.79	0.13
	AMED	86.42 ± 10.40	87.52 ± 13.41	0.08	3.36	− 0.09
	MED	86.78 ± 12.02	85.26 ± 14.40	0.16	2.07	0.11
	PMED	85.15 ± 15.45	84.40 ± 16.17	0.51	0.44	0.04
	POST	83.85 ± 13.61	83.35 ± 17.55	0.10	2.93	0.03
	PLAT	82.55 ± 15.47	79.52 ± 18.32	0.23	1.47	0.17
	LAT	78.15 ± 12.81	70.75 ± 16.53	0.11	2.71	0.5
	ALAT	83.67 ± 12.43	80.80 ± 16.75	0.09	3.07	0.19
Left post (cm)	ANT	87.85 ± 10.41	85.86 ± 12.66	0.18	1.87	0.17
	AMED	88.70 ± 11.75	86.06 ± 14.01	0.29	1.14	0.2
	MED	86.25 ± 13.11	87.05 ± 11.96	0.77	0.09	− 0.06
	PMED	87.75 ± 11.88	84.98 ± 15.25	0.21	1.63	0.2
	POST	85.95 ± 14.17	81.00 ± 17.23	0.10	2.77	0.31
	PLAT	84.19 ± 16.15	80.62 ± 17.23	0.56	0.34	0.21
	LAT	79.20 ± 13.10	72.00 ± 13.87	0.47	0.54	0.53
	ALAT	88.87 ± 13.17	84.99 ± 14.58	0.24	1.41	0.27

*ANT* anterior, *AMED* anteromedial, *MED* medial, *PMED* posteromedial, *POST* posterior, *PLAT* posterolateral, *LAT* lateral, *ALAT* anterolateral, *cm* centimeter, *d* effect size (Cohens).

Significant values are given in bold.

The results of the paired t-tests indicated significant improvements in both the SMFR) and MET groups across all measured outcomes post-intervention. In the SMFR group, the SRT improved from a pre-intervention mean of 7.29 ± 2.61 to a post-intervention mean of 9.32 ± 2.58, t = − 10.46, p < 0.001. In the MET group, the SRT improved from a pre-intervention mean of 5.78 ± 2.65 to a post-intervention mean of 7.84 ± 2.43, t = − 15.23, p < 0.001. For AKE-R, the SMFR group showed improvement from 43.50 ± 9.61 pre-intervention to 53.45 ± 8.98 post-intervention, t = − 6.70, p < 0.001, and the MET group improved from 43.40 ± 6.82 to 52.20 ± 5.91, t = − 6.29, p < 0.001. AKE-L also improved in the SMFR group, from 43.05 ± 8.13 to 54.05 ± 8.29, t = − 6.89, p < 0.001, and in the MET group, from 46.85 ± 8.37 to 55.65 ± 6.82, t = − 6.52, p < 0.001. Lastly, for the RMDQ, the SMFR group showed improvement from 12.65 ± 1.63 pre-intervention to 3.55 ± 1.05 post-intervention, t = 22.57, p < 0.001, and the MET group improved from 12.15 ± 1.46 to 3.85 ± 1.27, t = 31.61, p < 0.001.

**Figure 2:**
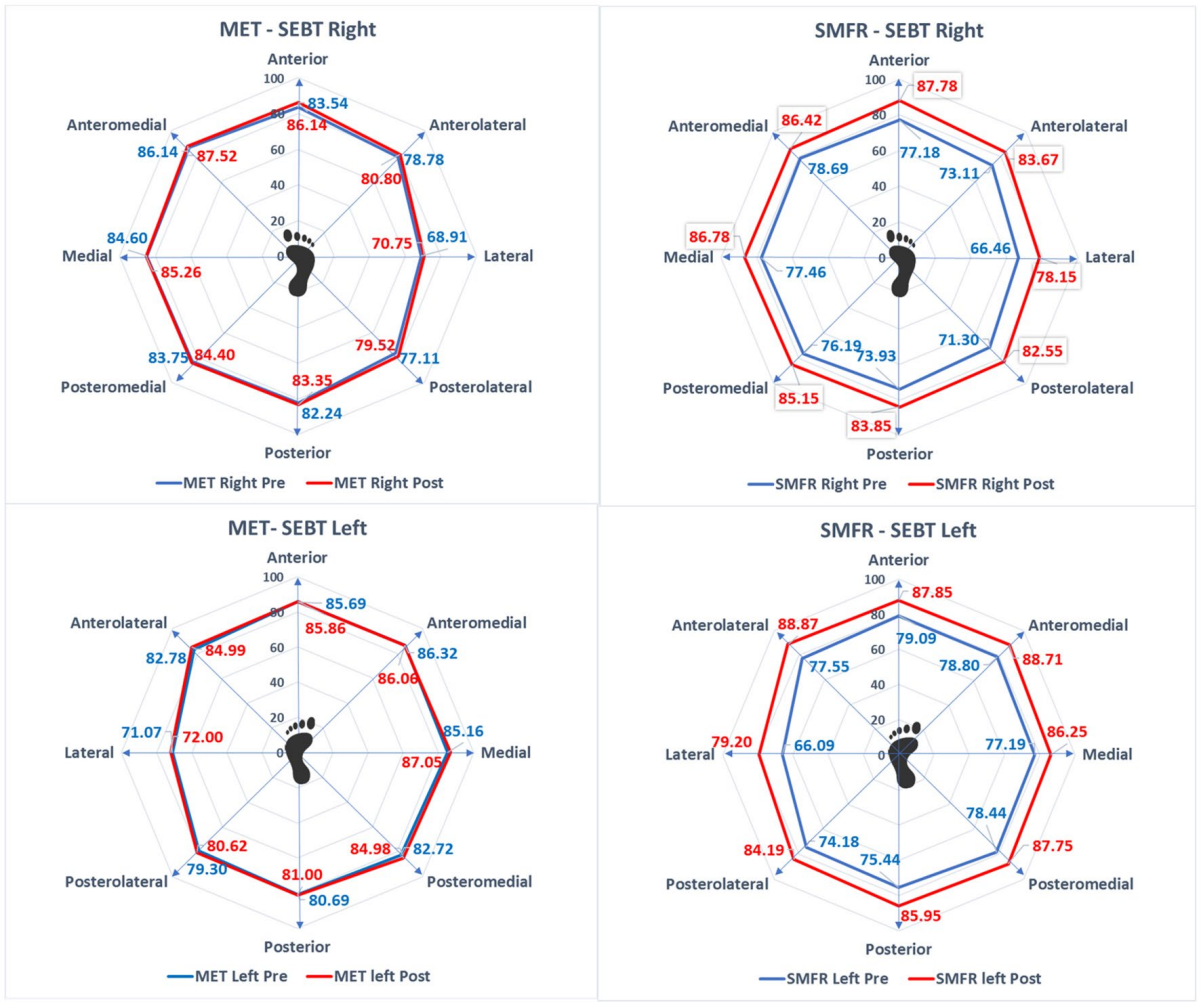
Star excursion balance test in the right and left leg following MET and MFR.

A paired t-test was performed to find the difference between pre- and post-measurement of dynamic balance using SEBT (right and left) following MET and SMFR. The assessment of dynamic balance before and after SMFR intervention was statistically significant for all directions of SEBT. However, there was no significant difference between pre- and post-measures of SEBT in different directions following MET for either the left or right leg (Fig. 2).

Table 3 shows the results of two way repeated measure ANOVA that participants in both the MET and SMFR groups had significant time effects (from pre- to post-assessment) in all the outcome variables like SRT, AKE-L, AKE-R, RMDQ, and SEBT. However, there was no significant group effect in any of the variables following the MET or SMFR interventions. Further, the time × group interaction was insignificant for SRT, AKE-L, AKE-R, and RMDQ. However, there was significant time x group interaction in the anterior, medial, lateral, and anterolateral directions of SEBT of the right leg and the anterior, anteromedial, posterior, and anterolateral directions of SEBT of the left leg.

**Table 3:** Two way repeated measure ANOVA table shows the time effect, group effect and time × group interaction in all the outcome variables, (95% CI, n = 40).

	Time effect			Group effect			Time x Group interaction		
	F	p	η^2^	F	p	η^2^	F	p	η^2^
SRT (cm)	299.5	p < 0.001	0.89	3.46	0.07	0.08	0.008	0.86	0.001
AKE-R (°)	99.53	p < 0.001	0.72	0.08	0.78	0.002	0.37	0.54	0.01
AKE-L (°)	89.67	p < 0.001	0.7	1.4	0.24	0.04	1.1	0.29	0.28
RMDQ	1307	p < 0.001	0.97	0.07	0.78	0.002	2.76	0.10	0.06
SEBT-R (cm)									
ANT	25.9	p < 0.001	0.4	0.44	0.51	0.12	9.5	0.004	0.2
AMED	14.8	p < 0.001	0.28	1.3	0.26	0.03	7.2	0.01	0.16
MED	13.5	p < 0.001	0.26	0.54	0.46	0.01	10.1	0.003	0.21
PMED	9.7	p < 0.001	0.2	0.5	0.48	0.01	7.3	0.01	0.16
POST	16.5	p < 0.001	0.3	0.63	0.43	0.01	10.5	0.02	0.21
PLAT	19.9	p < 0.001	0.3	0.06	0.70	0.002	8.3	0.006	0.18
LAT	19.1	p < 0.001	0.33	0.3	0.58	0.008	10.1	0.003	0.21
ALAT	23	p < 0.001	0.38	0.9	0.70	0.002	10.7	0.002	0.22
SEBT-L (cm)									
ANT	15.2	p < 0.001	0.2	0.43	0.51	0.01	14.1	0.001	0.27
AMED	9.2	p < 0.001	0.19	0.4	0.50	0.01	10.2	0.003	0.21
MED	13.9	p < 0.001	0.2	1.3	0.20	0.03	5.9	0.01	0.13
PMED	18.1	p < 0.001	0.03	0.03	0.86	0.001	6.7	0.01	0.15
POST	12.4	p < 0.001	0.2	0.001	0.97	0.10	11	0.002	0.22
PLAT	7.6	p < 0.009	0.16	0.2	0.86	0.001	4.5	0.40	0.1
LAT	23.9	p < 0.001	0.3	0.7	0.70	0.002	18	0.102	0.3
ALAT	22	p < 0.001	0.3	0.25	0.87	0.001	9.9	0.003	0.2

*SRT* sit and reach test, *AKE-R* active knee extension of right leg, *AKE-R* active knee extension of left leg, *RMDQ* Roland–Morris disability questionnaire, *cm* centimeter, *ANT* anterior, *AMED* anteromedial, *MED* medial, *PMED* posteromedial, *POST* posterior, *PLAT* posterolateral, *LAT* lateral, *ALAT* anterolateral.

## Discussion

Two-wheeler riders often adopt particular postures when riding that might exacerbate spinal issues and, by extension, impair day-to-day functioning. Ergonomic posture, particularly motorcycle riding, is vital in CLBP management and prevention. Proper alignment during riding distributes body weight evenly, reducing lumbar spine strain and mitigating pain triggers. Prior research shows that between 10 and 60% of riders adopt a flexed posture when riding, increasing their risk of lumbar spine injury^23^. They also showed a correlation between the angles and positions of the rider's joints and their range of motion. The purpose of the present study was to examine the efficacy and to compare the 4 weeks of Muscle Energy Techniques (MET) and (self-myofascial release) SMFR technique using a foam roller on hamstring flexibility, dynamic balance, and physical disability in two-wheeler riders with CLBP. In exploring therapeutic interventions for C LBP, this study highlights the significant roles of MET and SMFR. Flexibility of the hamstrings and lumbar spine was assessed using the sit and reach test (SRT). The active knee extension test (AKE) was used to evaluate the length of the hamstring muscle. The length of the hamstrings has been linked to a shift in lordosis and an increased risk of leg injury^24,25^.

Both MET and SMFR interventions showed significant changes in the hamstrings and lumbar spine flexibility, and physical disability from the baseline measurements. The RMDQ showed that there was almost a 75% improvement in the disability level following both intervention groups. Patel R et al. (2019) conducted a one-time study to show that foam rolling and MET are equally effective in improving hamstring flexibility measured by SLR and AKE tests^18^. Peacock et al. investigated the effects of foam rolling along two different axes of the body in conjunction with a dynamic warmup on male volunteers. Foam rolling along the mediolateral axis increased sit and reach scores substantially more than rolling along the anteroposterior axis^26^. Foam rolling's effects on knee flexion range of motion (ROM) and quadriceps neuromuscular activation were studied by MacDonald et al. When comparing the foam rolling group to the control group, the authors discovered a statistically significant increase in knee flexion ROM between baseline measurement and post-test^27^. The results found in the flexibility and disability of the hamstring muscles of patients treated with SMFR using a foam roller may be attributed to an immediate reduction in arterial stiffness and improved endothelial vascular function, which promote optimum blood flow^28^. One of the most popular ways to lessen injury risk is using foam roller treatments, which break stuck myofascial fibers^29^.

SMFR offers a unique approach to alleviating CLBP by focusing on the fascia, the connective tissue surrounding muscles. Through sustained pressure using a foam roller, SMFR aids in breaking down fascial adhesions and improving tissue mobility. This process not only helps in reducing pain but also contributes to improved range of motion and muscle function, which are often compromised in CLBP sufferers. Fama and Bueti suggested that pressure exerted by foam rolls stimulates the Golgi tendon unit and reduces muscle tension^30^. Another potential benefit is enhanced tissue hydration, which occurs when soft tissue is compressed like a sponge during treatment. This increases blood flow and temperature and improves motion between the fascial layers. By affecting pain-modulatory systems, foam rollers may improve analgesic effects and muscle rehabilitation^29,31^.

Furthermore, the foam roll enhances muscle recovery and improves its performance. Following SMFR, there was a significant change in all the directions of the SEBT for the left and right leg. However, the MET group did not have any significant difference between pre and post-measures of the SEBT for both the left and right leg in all directions. Due to the high-density free nerve endings found in the fascia, part of the sympathetic nervous system, a more relaxed parasympathetic response is displayed. Additionally, Ruffini corpuscle activity is seen due to the heat generated by the foam roller and soft tissue stimulation, which improves dynamic balance ability and range of motion^32–35^.

On the other hand, MET, a form of manual therapy, targets muscular imbalance and joint dysfunction primarily by utilizing isometric contractions to improve muscle strength and flexibility. This technique addresses the underlying issues of muscle tightness and imbalance contributing to CLBP. By engaging the patient in active participation, MET enhances muscular function and promotes body awareness, leading to more effective management of CLBP. It has been found that the shortened hamstring muscle of the patients treated with MET has improved flexibility due to autogenic inhibition, which occurs due to the inhibitory Golgi tendon reflex. This reflex is thought to be triggered by isometric muscular compression, which ensures that the Golgi tendon organs are stretched, and the muscle relaxes reflexively^36^. In addition, Handel et al., reported using a post-isometric extending approach, such as MET, that resulted in significant changes in range of motion and hamstring muscle extensibility compared to static or ballistic extending^37^. In a study of university males, MET was found to be more effective than eccentric exercise in developing hamstring flexibility^38^. MET was found to be more significant in enhancing hamstring flexibility than eccentric preparation in a study of university males. Wilson E et al. support the benefits of MET on disability. According to his study, MET has been proven to be useful in reducing back pain and disability in patients with acute LBP when combined with neuromuscular re-education and resistance training^39^. In another trial, MET was found to be beneficial in reducing physical disability^40^. Our findings suggest an improvement in pain and physical disability, supporting this research finding.

It should be emphasized that SFMR using a foam roller or roller massager is a burgeoning field of study that has not yet achieved its apex and that our analysis is limited by the questions and search criteria used. In the study that met the inclusion criteria, diverse methodologies and outcome measures hampered direct comparisons and building a consensus regarding the best program. The study was confined to CLBP from nonspecific and non-traumatic conditions and did not include riders carrying heavy weights on their shoulders.

The study had few limitations. Firstly, it predominantly focused on a specific demographic within a limited age range and did not include riders carrying heavy weights, potentially limiting the generalizability of the findings. Secondly, the study's assessment was primarily centered on changes in hamstring flexibility, dynamic balance, and physical disability, neglecting other relevant outcome measures such as pain intensity or long-term effects. Additionally, the absence of a control group makes it challenging to attribute observed improvements solely to the interventions. The study's short four-week follow-up duration might not capture the sustainability of benefits over time.

Future studies should investigate the changes in other muscle lengths and ergonomic findings. A follow-up study on different demographics, such as teenagers, older adults, women, and other athletes, with the application time, duration, and diversity of SMFR applications could yield exciting results. Both MET and SMFR can be used in clinical practice for treating the tight hamstring of bikers with CLBP. MET can only be done in the clinical setup under the observation of required professionals, but SMFR can be done by oneself at home.

## Conclusions

Both Self MFR using a foam roller (SMFR) and (muscle energy technique) MET are effective in improving the hamstring muscle flexibility, dynamic balance, and physical disability in two-wheelers with CLBP, however, SMFR was found to be more effective than MET.

## Data Availability

The data presented in this study are available on request from the corresponding author. The data are not publicly available due to privacy restrictions.
